# Does Older Adults’ Cognitive Function Disrupt the Malleability of Their Attitudes toward Outgroup Members?: An fMRI Investigation

**DOI:** 10.1371/journal.pone.0152698

**Published:** 2016-04-13

**Authors:** Anne C. Krendl, Elizabeth A. Kensinger

**Affiliations:** 1 Department of Psychological and Brain Sciences, Indiana University, Bloomington, IN, United States of America; 2 Department of Psychology, Boston College, Chestnut Hills, MA, United States of America; Center for BrainHealth, University of Texas at Dallas, UNITED STATES

## Abstract

In the current study we examine how individual differences in older adults’ global cognitive function impacts the extent to which their attitudes toward stigmatized individuals are malleable. Because prior research has elucidated the neural processes that are involved in evaluating stigmatized individuals who are responsible or not responsible for their condition, a cognitive neuroscience approach may be well-suited to answer this question. In the current study, 36 older and 17 young adults underwent functional magnetic resonance imaging while evaluating images of homeless people who were described as being responsible or not responsible for their condition. They also indicated how much pity they felt for each of the individuals in order to determine the extent to which their attitudes were malleable (e.g., more pity for not-responsible as compared to responsible individuals). Participants’ cognitive function and baseline measure of their attitudes toward stigmatized individuals (including homeless individuals) were assessed. Results revealed that although older adults’ attitudes were malleable, the extent to which this was true varied due to individual differences in their global cognitive function. Specifically, the difference in the magnitude of older adults’ self-reported pity for not-responsible as compared to responsible homeless individuals was predicted by their global cognitive function. Moreover, the difference in pity that older adults expressed toward not-responsible as compared to responsible homeless individuals was related to activity in the left insula and the anterior cingulate cortex (regions implicated in empathy). These results suggest that attitude malleability is affected by individual differences in global cognitive function.

## Introduction

Recent research suggests that individual differences in older adults’ cognitive function exacerbates their expression of negative bias to certain outgroup members [1,2,3,4]. Age-related declines in regulatory ability have been implicated as the primary reason for older adults’ increased bias [1,2,3,4,5], which is consistent with prior research demonstrating that regulatory ability allows people to actively inhibit stereotypic responses and prejudice [1,4,6,7] in order to behave non-prejudicially [8,9]. An important caveat to these findings is that they have primarily focused on older adults’ existing bias toward outgroup members [1,2,3,4], largely overlooking whether older adults’ bias is malleable.

Prior research suggests that young adults’ bias toward stigmatized individuals is somewhat mitigated when they are told that the stigmatized individual is not responsible for the onset of his or her condition. Specifically, the less responsible stigmatized individuals are perceived as being for their condition, the more pity they elicit from perceivers [10,11,12,13,14,15]. The extent to which individuals feel more pity for a stigmatized individual depicted as being not responsible as compared to responsible for his or her condition reflects how malleable their attitudes are. Emerging research in the field of social neuroscience has identified regions that are more active when young adults evaluate stigmatized individuals who are perceived to be less responsible for the onset of their condition [16,17,18,19]. These studies have shown that participants have greater activity in neural regions associated with empathy (e.g., insula and anterior cingulate cortex; [17,18,19] and regulatory effort (e.g., right ventrolateral prefrontal cortex) [16,17,19] when they evaluated stigmatized individuals who were depicted as not being responsible for the onset of their condition.

Behavioral research, however, suggests that older adults hold non-stigmatized individuals more responsible for their actions than do young adults [20,21,22] and are less susceptible to responsibility manipulations when evaluating stigmatized individuals (suggesting their attitudes are less malleable), particularly when they have experienced declines in their global cognitive function [23]. One possibility as to why that might be is that manipulations of perceived responsibility require numerous different cognitive and affective processes–e.g., inhibition, working memory, and empathy [19]–which are disrupted with healthy aging [24,25,26]. Thus, older adults who have experienced overall declines in their cognitive abilities may be less effective in changing their negative affective response to stigmatized individuals in manipulations where the perceived responsibility of the stigmatized target is altered. The current study therefore used a neuroimaging approach to examine why older adults with impaired global cognitive function have less malleable negative affective responses toward stigmatized individuals (e.g., less difference in the amount of pity they express toward stigmatized individuals who are portrayed as being not-responsible as compared to responsible for their condition).

Empathy and regulatory ability are both disrupted with age. Empathy–an ability to share the feelings of others–is associated with heightened activation in both the insula and anterior cingulate cortex [27]. Individual differences contribute to the extent to which perceivers experience empathy [28], and previous research has demonstrated that certain aspects of empathy are impaired with age [24]. As previously discussed, older adults with impaired global cognitive function also are less successful in inhibiting their bias toward outgroup members [1,2,3,4,5]. For instance, Gonsalkorale and colleagues [5] demonstrated that older adults’ anti-age bias was attributed to their deficits in inhibiting the expression of stereotypes. With these findings in mind, older adults with more impaired global cognitive function may have less malleable attitudes toward stigmatized individuals who are perceived to be responsible for the onset of their condition either because they have less empathy for those individuals, because they engage less regulatory effort, or a combination of both.

One limitation to using a strictly behavioral approach to disentangle these possibilities is that they all make the same prediction–as compared to older adults with relatively preserved cognitive function, older adults with relatively impaired global cognitive function have less malleable attitudes toward stigmatized individuals who are described as being responsible (vs. not responsible) for the onset of their condition. However, because the neural correlates engaged in dissociating perceptions of accountability have previously been proposed [16,17,18,19], neuroimaging can provide insight into the mechanisms underlying the effects of individual differences in global cognitive function on older adults’ ability to dissociate perceived accountability in stigmatized individuals. Correctly identifying these mechanisms is important for developing interventions that are effective in reducing older adults’ bias. Specifically, if older adults with more impaired global cognitive function have less empathy for stigmatized individuals, then they should have less activity in the insula and anterior cingulate cortex, regions that have been consistently implicated in experiencing empathy [27]. Alternatively, if older adults with more impaired global cognitive function engage less regulatory effort when evaluating stigmatized individuals, then we should observe relatively reduced activation in the lateral prefrontal cortex, a region that has been widely implicated in regulatory effort [16,19]. Finally, if both processes are disrupted, then we would anticipate a combination of activation differences among the empathy and regulatory regions.

Since previous research has demonstrated that individual differences in older adults’ global cognitive function impacts their preexisting notions of responsibility in stigma [23], we explicitly manipulated the perceived responsibility that the stigmatized individual was depicted as having for his or her condition in order to assess older adults’ attitude malleability. Prior to completing this task, we measured participants’ explicit bias in order to control for individual differences in bias in our analyses. This approach was necessary because if older adults with relatively impaired global cognitive function have more bias toward stigmatized individuals than those with relatively preserved global cognitive function, this could disrupt the extent to which they can (or will) change their affective responses to stigma. Since our goal was to determine whether individual differences in older adults’ cognitive function was associated with a reduction in the malleability of their attitudes toward stigmatized individuals irrespective of their explicit bias, it was therefore important to control for this bias.

We used a similar approach to the fMRI study by Krendl, Moran, and Ambady [19]. The two critical differences in the current study were that we measured participants’ explicit bias toward homeless individuals, and we asked participants to indicate how much pity they felt for each stigmatized individual in order to assess how malleable their attitudes were. This approach allowed us to quantify the amount of pity that individuals expressed toward stigmatized individuals, as well as to relate those differences to neural activity. We also measured individual differences in empathy [28] in order to determine whether empathy (and related behaviors such as altruism) might underlie observed differences. We hypothesized that older adults with more impaired global cognitive function would have less malleable attitudes (e.g., a smaller difference in the amount of pity they expressed for a stigmatized individual who as described as being not responsible as compared to responsible for his or her condition), which would replicate prior work by Krendl and Wolford [23]. We anticipated that this would emerge when controlling for individual differences in explicit bias, suggesting that attitude malleability is a distinct (although likely overlapping) construct from bias. Importantly, we anticipated that reduced attitude malleability would be because they have less empathy for those individuals and engage less regulatory effort when evaluating them.

## Methods

A total of 36 older adults (M_*age*_ = 72.2 years, SD = 6.5 years, 17 female) and 17 young adults (M_*age*_ = 20.3 years, SD = 1.2 years, 10 female) who were right-handed and without a neurological, cardiovascular, or psychiatric diagnosis were recruited from the greater Boston area to participate in this study. One older adult and two young adults were Asian, the rest were White. Both older and young adults participated for $25/hour. All participants provided written informed consent. The consent procedure and study were approved by the Institutional Review Boards at Indiana University and Boston College. Anatomical and functional whole-brain imaging was performed on a 3.0T Siemens Trio Scanner (Trio, Siemens Ltd., Enlargen, Germany) using standard data acquisition protocols. Anatomical images were acquired using a high-resolution 3-D magnetization prepared rapid gradient echo sequence (MP-RAGE; 144 sagittal slices, TE = 7 ms, TR = 2200 ms, flip angle = 7^0^, 1 x 1 x 0.89 mm voxels). Functional images were collected in three functional runs: two consisting of 171 time points each, and one consisting of 132 times points each. For all three runs, we used a fast field echo-planar sequence sensitive to blood-oxygen level-dependent contrast (T2*) (36 axial slices per whole-brain volume, 3mm in-plane resolution, 4mm thickness, 0mm skip, TR = 2500ms). At the start of each functional scan, three dummy scans were acquired to allow for stabilization of the fMRI signal. These scans were discarded and not included in any of the analyses.

### Behavioral tasks

#### Assessing cognitive function

Older adults (OA) were recruited for the current study based on their performance on five diagnostic measures to assess global cognitive function: Wisconsin Card Sorting Task (WCST), FAS word fluency, WAIS-R mental arithmetic, WMS-R mental control, and WMS-R backward digit span [29]. These measures have been used in previous studies to assess the effects of individual differences in older adults’ global cognitive function on their expression of bias toward outgroup members [2,23,30]. Two participants had completed these measures in a separate testing session within a year of the fMRI session, the remaining older adults completed the battery within approximately three months of undergoing fMRI. Performance on each measure was z-scored and weighted according to Glisky and colleagues [29], resulting in one global cognitive function value per participant. We selected two groups of OA from a pool of approximately 200 who had previously completed the global cognitive function measures described above: OA who performed relatively well on the cognitive tasks (17 OA with relatively preserved cognitive function; M_*score*_ = 0.50 SDs above the mean, SD = 0.24; M_*age*_ = 74.8 years, SD = 5.3 years, 4 female), and those who performed relatively poorly on the tasks (15 OA with relatively impaired cognitive function; M_*score*_ = -0.50 SDs below the mean, SD = 0.37; M_*age*_ = 74.3 years, SD = 6.0 years, 10 female). A t-test confirmed that OA with relatively preserved global cognitive function score was significantly higher than OA with relatively impaired cognitive function’s scores, *t*(30) = 9.14, *p* < .001. Due to logistical constraints, young adults (YA) were administered the global cognitive function battery on the day of the scan. Overall, YA’s global cognitive function scores were not significantly different from OA with relatively preserved global cognitive function scores (M_*YA score*_ = 0.62 SDs above the mean, SD = 0.43; *t*(31) = 1.01, *p =* .32). For this reason, differences attributable to global cognitive function were evaluated by comparing the two groups of older adults, whereas differences attributable to age were evaluated by comparing all of the young adults to all of the older adults. All OA and YA in the current study scored above 26 on the Mini-Mental State Exam [31], suggesting that they were normal functioning [32].

#### Pre-test surveys

In addition to completing the cognitive tasks, all but one of the OA (an OA with relatively preserved cognitive function) also completed the Shipley vocabulary test. Overall, OA with relatively preserved cognitive function outperformed OA with relatively impaired cognitive function on this measure (OA with relatively preserved global cognitive function = 37.25 words, SD = 1.88; OA with relatively impaired global cognitive function = 34.10 words, SD = 3.22; *t*(28) = 3.50, *p* < .005). This finding was consistent with their self-reported overall years of education (OA with relatively preserved global cognitive function = 16.07 years, SD = 2.12; OA with relatively impaired global cognitive function = 14.17 years, SD = 2.08; *t*(25) = 2.15, *p* < .05). However, OA with relatively preserved global cognitive function and OA with relatively impaired global cognitive function did not differ in their self-reported mood (as measured by the Geriatric Depression Scale), nor did they differ in their performance on the MMSE (both *ts <* 1).

Prior to the scanning session, participants completed a series of surveys. The surveys included the shortened 22-item Empathy Quotient (EQ) developed by Wakabayashi and colleagues [28], and a modified version of the Self-Report Altruism Scale [33]. The Self-Report Altruism scale was modified by including two additional items that assessed participants’ altruism toward homeless individuals: “I have given money or goods to a homeless person” and “I have volunteered at a homeless shelter”. These items were in addition to the existing 20 items that assessed altruism more generally (e.g., “I have donated blood” and “I have given money to a charity”).

#### Imaging task

During the scanning session, participants completed two separate tasks. First, participants completed a person-rating task in which they viewed 30 images of extreme stigmatized individuals (homeless individuals and substance abusers) [34], 30 images of other stigmatized individuals (amputees and individuals with facial deformities), and 30 images of non-stigmatized control images. Half the control images were matched in age, gender, and pose to the extreme stigmatized individuals, the other half were matched in age, gender, and pose to the other stigmatized individuals. With respect to pose, control images were matched to the extreme stigmatized images to include images of individuals lying down or slouched (which was similar to the homeless images we used), or holding an object (e.g., coffee cup), which was similar to the substance abuser images we used. The full body shot control images for the other stigmatized images featured images of non-amputee individuals walking or sitting with their limbs visible (which paralleled the amputee images) and face-only shots of individuals without facial deformities (as controls for the facial deformity images). These images were similar to those that were used in previous research examining age differences in stigma perception [2]. Participants viewed each image for 2.5 seconds, during which time they also indicated via button press (with their right hand) whether they thought they would like or dislike the person in the image, which provided a baseline measure of their explicit bias which were used in the present analyses (see Results). Each image was presented for 2.5 seconds in an event-related fashion, with jittered fixation throughout ranging from 0–7.5 seconds (M_*jitter*_ = 3.4 seconds, SD = 1.6 seconds). The fMRI data from this task were not used in the current study, and will not be considered further.

Second, following approximately 15-minutes of structural scanning, participants completed a first-impression task in which they viewed a series of short sentences describing someone who became homeless (described below). Each sentence was presented for 5 seconds and was followed by a variable ISI of 0–7.5 seconds. Participants then saw a picture of a homeless person for 2.5 seconds. The images used in this task were different from the images used on the person-rating task. Participants were instructed to use the sentence to form an impression of the homeless individual paired with it. Specifically, they were instructed to indicate via button press (with their right hand) how much pity they felt for the individual it described (1 = no pity, 4 = very much pity) when his or her image appeared on the screen. Participants completed several practice trials to ensure they understood the directions and could respond to each image in 2.5 seconds. ITI ranged from 0–7.5 seconds (ISI: M_*jitter*_ = 2.5 seconds, SD = 2.0 seconds; ITI = M_*jitter*_ = 3.6 seconds, SD = 2.8 s seconds). On any given trial, both the sentence and the picture were unique (that is, the images of homeless individuals were not repeated for the same participant, although the same set of images were presented across all the participants). The conditions in which each picture was presented (e.g., responsible or not-responsible) varied across participants.

At the conclusion of the MRI study, participants rated the 60 images of homeless individuals that they had viewed during the evaluation task on valence (1 = very negative, 7 = very positive) and arousal (1 = extremely weak emotional intensity, 7 = extremely strong emotional intensity). Individual ratings of valence and arousal were used as regressors in subsequent imaging analyses (see below).

#### Stimuli and pilot testing

The images of homeless individuals used in the current study were collected from Internet search engines (e.g., google images) using search terms such as “homeless.” Many of the images were used in prior research [2,19]. All of the homeless individuals were White, depicted alone, and ranged in age (from young adult to older adult). Each image was modified in Adobe Photoshop to a standard height of 360 pixels with a resolution of 72 pixels/inch. Each image depicted only one individual who was the central element of the photo, and were a subset of the images as those used by [19].

We presented participants with a total of 60 sentences, each paired with a unique picture of a homeless individual: 30 described an onset that depicted the individual as being responsible for his homelessness (e.g., “lost his money after he was caught embezzling”), while the other 30 described an onset where the individual was depicted as not being responsible for his homelessness (e.g., “lost his job when his company downsized”). The 60 sentences were selected from pilot testing conducted with a different group of older adults than those in the current study. The participants rated the sentences on perceived responsibility (i.e., “1 = the person described is not at all responsible for becoming homeless; 7 = the person described is extremely responsible for becoming homeless”), as well as valence and arousal on a 1 to 7 Likert scale (1 = extremely negative or low arousal, 7 = extremely positive or high arousal). The individuals described in the responsible onset scenarios were rated as being significantly more responsible for becoming homeless (M_*responsible*_ = 6.57, SD = 0.31) than those described in the not-responsible onset scenarios (M_*responsible*_ = 2.71, SD = 0.56). However, the responsible and not-responsible scenarios did not differ in valence or arousal (responsible: M_*valence*_ = 3.14, SD = 0.52; M_*arousal*_ = 5.03, SD = 0.46; not-responsible: M_*valence*_ = 3.09, SD = 0.53; M_*arousal*_ = 5.04, SD = 0.57). The two scenarios also did not differ in their respective word length (responsible: M_*length*_ = 10 words, SD = 2.33; not-responsible: M_*length*_ = 9.67 words, SD = 1.79). Further, sentence and image pairs were pseudorandomized such that approximately half the participants saw 30 images of homeless individuals paired with responsible onset scenarios, whereas the remainder of the participants saw those same images paired with not-responsible onset scenarios.

### Data analysis

All fMRI data were analyzed using the general linear model for event-related designs in SPM8 (Wellcome Department of Cognitive Neurology, London, UK). Functional data were coregistered to individual structural volumes, and underwent standard preprocessing (e.g., slicetime correction, motion correction, unwarping, and spatial realignment) to remove sources of noise and artifact. Functional data were also spatially smoothed (8mm full-width-at-half-maximum [FWHM]) using a Gaussian kernel. Using custom artifact detection software to detect motion artifact (http://www.nitrc.org/projects/artifact_detect), individual runs were analyzed on a participant-by-participant basis to find outlier time points after preprocessing. Specifically, this toolbox allowed us to identify volumes during which participant head motion exceeded 1 mm and volumes in which the overall signal for that time point fell three SDs outside the mean global signal for the entire run. Outlier time points were then entered as nuisance regressors in the GLM analysis via the use of participant-specific regressors of no interest. For all participants, less than one percent of the data was included as a nuisance regressor. A one-way ANOVA on the percentage of nuisance regressors with participant group (YA, OA with relatively preserved global cognitive function, and OA with relatively impaired global cognitive function) as the between-subject variable revealed no significant effect of participant group (*F*(2,46) = 2.50, *p* = .09). Three older adults and one young adult were excluded from subsequent analyses because they moved more than 3mm during each functional run. One additional older adult was excluded from related behavioral and fMRI analyses for not complying with task instructions.

FMRI analyses were conducted using a general linear model (GLM). The GLM incorporated task effects for the two different image types (images paired with a responsible onset and images paired with a not-responsible onset), as well as the sentences (which conveyed the responsible onset or not-responsible onset information) along with covariates of no interest (a session mean, a linear trend to account for low-frequency drift, six movement parameters derived from realignment correction, and individually defined motion artifacts). The GLM was convolved with a canonical hemodynamic response function and used to compute contrast images (containing weighted parameter estimates) for each comparison at each voxel and for each subject.

We also conducted a parametric modulation by collapsing across stigma acquisition (responsible or not-responsible) and using the individual ratings of pity for each image as the covariate of interest (modeled linearly), while simultaneously regressing out covariates of no interest (participants’ post-test valence and arousal ratings in response to each image of a homeless individual, collapsed across stigma acquisition). The separate parametric regressors for the participants’ post-test valence and arousal ratings in response to each homeless individual image allowed us to control for any variability in the neural response that was due to individual or age differences in the emotional intensity of the images. The parametric modulation modeled a linear increase in the hemodynamic response that paralleled the linear increase in individual ratings of pity. Each participant’s behavioral response (a numerical response on a scale of 1 to 4) was scored such that a score of 1 would be equivalent to a “low pity” rating, and 4 would be a “high pity” rating.

Region of interest (ROI) analyses were conducted using the functional ROIs tool in SPM8 (marsbar; http://marsbar.sourceforge.net/). For all ROI analyses, we averaged the parameter estimates from the 8mm sphere surrounding the peak coordinate of interest. The peaks we used for the ROI analyses were identified by using the contrast from the conditions of interest (e.g., not-responsible > responsible) relative to baseline fixation. In order to acquire unbiased ROIs for additional analyses, we identified peaks from Neurosynth (http://neurosynth.org) [35]–an online database that conducts meta-analyses by regions or topics based on existing neuroimaging research. Consistent with Krendl, Moran, and Ambady [19], we examined the results for the impression formation task at a threshold of *p* < .005 uncorrected. In order to calculate the corrected *p* < .05 threshold, we used a Monte Carlo conversion script from [36] to determine the extent threshold required to convert *p* < .005 uncorrected to *p <* .05 corrected. We used 1000 iterations of the Monte Carlo to ensure an accurate estimation for the extent threshold.

## Results

We had two questions in our analyses. First, we examined whether age uniquely affected behavioral and neural responses to stigmatized individuals. Second, we examined whether global cognitive function uniquely contributed to participants’ behavioral and neural responses to stigmatized individuals. For effects associated with age, we compared YA overall to OA overall. However, for effects associated with global cognitive function, we compared OA with relatively preserved global cognitive function to OA with relatively impaired global cognitive function. We only compared the OA here because the two groups of OA differed in their respective levels of global cognitive function, whereas OA with relatively preserved global cognitive function scores did not differ from YA. An additional benefit of comparing the two samples of older adults (e.g., individuals with relatively impaired and individuals with relatively preserved cognitive function) in this way is that it controls for potential cohort differences in task performance or decision-making that could otherwise confound the results.

### Behavioral results

#### Survey results

We first examined whether age influenced performance on the Empathy Quotient [28] and the Self-Report Altruism Scale [33]. Although YA and OA did not significantly differ in their self-reported empathy (*t*(43) = 1.73, *p =* .09), OA had higher altruism as compared to YA (*t*(46) = 2.05, *p <* .05). Self-reported empathy and altruism did not differ as a function of global cognitive function (both *ts* < 1, *ps >* .38). See Table 1 for all means, and SDs.

**Table 1:** Mean Empathy Quotient and altruism ratings for young adults (YA) and older adults (OA).

Scale	YA	OA with relatively preserved global cognitive function	OA with relatively impaired global cognitive function
Empathy Quotient	28.07 (8.15)	25.18 (11.18)	25.00 (6.93)
Altruism	37.80 (9.06)	45.42 (7.55)	42.53 (9.61)

SD ().

#### Does age and/or global cognitive function predict participants’ pity differential toward homeless individuals?

We next examined whether OA and YA differed in the malleability of their attitudes toward homeless individuals in the not-responsible as compared to the responsible onset condition. In order to do this, we calculated a pity differential score for each participant (e.g., the difference in the amount of pity they expressed toward the homeless individuals in the responsible v. not-responsible conditions). An important consideration in this analysis, however, is whether individual differences in participants’ baseline explicit attitudes could underlie any differences that emerged in the pity differential. We therefore entered these attitudes as a covariate. Results revealed no significant difference between the two participant groups, *F <* 1, *p* = 0.45.

We next examined whether global cognitive function affected the pity differential. Here, in addition to controlling for baseline explicit attitudes, we also controlled for differences in years of education (which was correlated with older adults’ global cognitive function: *r*(31) = .48, *p* < .001) using a hierarchical multiple regression, with pre-existing attitudes and years of education entered together (in that respective order) in the first step, and global cognitive function added to the second step. Results revealed that although the model was not significant at the first step: *F*(2,29) = 2.52, *p* = .10, explicit attitudes did significantly predict pity differential (see Table 2 for means). However, adding global cognitive function (as measured by their performance on a battery of cognitive tasks) [29] in the second step significantly accounted for 31% of the variance in the pity differential score: (*F*(3,29) = 3.96, *p* < .02; Δ*F*(1,26) = 5.92, *p* = .02). In other words, higher global cognitive function was associated with greater differentiation in the amount of pity that older adults expressed toward homeless individuals as a function of the perceived onset of their condition (e.g., not-responsible or responsible). Regression statistics are reported in Table 3.

**Table 2:** Mean pity ratings for young adults (YA) and older adults (OA).

Onset type	YA	OA with relatively preserved global cognitive function	OA with relatively impaired global cognitive function
Responsible onset	1.73 (0.58)	1.48 (0.29)	1.59 (0.63)
Not responsible onset	3.44 (0.51)	3.50 (0.52)	3.36 (0.49)

SD ().

**Table 3:** Summary of Hierarchical Regression Analysis Predicting Older Adults’ pity differential score.

Variable	ß	*t*	*R*	*R*^*2*^	Δ*R*^*2*^
Step 1			.40	.16	.
Explicit attitudes	-.39	-2.21*			
Years of education	.04	.25			
Step 2			.56	.31	.16
Explicit attitudes	-.55	-3.15**			
Years of education	-.20	-1.04			
Global cognitive Function	.49	2.43*			

*p < .05.

** *p <* .01.

### fMRI results

#### Using fMRI to identify differences in the neural mechanisms underlying participants’ pity differential

The behavioral results identified no behavioral differences in the pity differential as a function of age, but we did identify differences that were associated with OA’s global cognitive function. The neuroimaging results examined why that might be. We hypothesized that older adults with more impaired global cognitive function had a lower pity differential (e.g., less malleable attitudes) because they have less empathy for those individuals and engage less regulatory effort when evaluating them. There were therefore three goals of the neuroimaging analyses, each of which built on the prior. First, we sought to identify the neural regions involved in attitude malleability for each of the three groups. Here, we conducted a whole-brain ANOVA comparing across all three groups in order to provide a more stringent test of our hypotheses. Of interest in this analysis was to identify the group differences that emerged in these regions. Second, we isolated the regions that differed by group that were involved with the construct of interest: the pity differential. Specifically, we identified which of the regions from main effect of group (from the first analysis) were related to pity. Finally, we determined the extent to which the pity differential was associated with differences in empathy (e.g., insula and anterior cingulate cortex) or regulatory effort (e.g., right ventrolateral prefrontal cortex) when controlling for individual differences in their explicit bias. These questions are each discussed in separate sections below.

#### Question 1: Whole-brain ANOVA: Are there group differences in the neural regions involved in attitude malleability?

In the fMRI analyses, the pity differential was first examined by identifying neural regions that differed as a function of the manipulation (not-responsible v. responsible) and group (young adult, OA with relatively preserved global cognitive function, OA with relatively impaired global cognitive function). We thus entered these variables into a whole-brain ANOVA. Results revealed no effect of responsibility or interaction, but a main effect of group in several brain regions, including: right cuneus (BA 19: 6, -90, 45), right cerebellum (57, -60, -33), right superior frontal gyrus (BA 8: 24, 45, 54), bilateral parietal sulcus (right BA 7: 30, -60, 48, left BA 7: -27, -57, 66), bilateral fusiform gyrus (left BA 20: -48, -27, -18, right BA 37: 48, -57, 3), right anterior cingulate gyrus (BA 32: 15, 24, 36), bilateral ventrolateral prefrontal cortex (right BA 47: 45, 30, -15; right BA 44: 42, 21, 15; left BA 47: -51, 18, -18; left BA 44: -60, 6, 21), and right middle frontal gyrus (BA 9: 24, 42, 27). For complete list of activations, see Table 4.

**Table 4:** Statistically significant clusters from the main effect of group in the voxel-wise whole brain ANOVA. —indicates a region that does not fall within a Brodmann area.

	MNI Coordinates	Brodmann area	F	cluster
Right cuneus	6, -90, 45	19	40.69	1645
Right cerebellum	57, -60, -33	-	15.46	35
Right superior frontal gyrus	24, 45, 54	8	15.51	416
Right superior parietal cortex	30, -60, 48	7	14.83	52
Left fusiform gyrus	-48, -27, -18	20	12.36	269
Right precentral gyrus	51, -6, 63	4	12.27	133
Right precentral gyrus	42, 6, 24	6	11.44	33
Left cerebellum	-30, -42, -12	-	11.36	18
Right middle frontal gyrus	6, 3, 63	6	11.33	35
Right superior temporal gyrus	69, -21, 18	22	11.06	91
Left cerebellum	-24, -63, -30	-	10.47	103
Thalamus	0, -3, 9	-	10.42	53
Right insula	39, -15, -6	-	9.62	27
Midbrain	9, -21, -27	-	9.61	39
Right anterior cingulate cortex	15, 24, 36	32	8.98	41
Right cerebellum	33, -39, -15	-	8.82	26
Right cingulate gyrus	18, 42, -6	32	8.67	21
Right ventrolateral prefrontal cortex	45, 30, -15	47	8.61	29
Left ventrolateral prefrontal cortex	-51, 18, -18	47	8.29	33
Left ventrolateral prefrontal cortex	-60, 6, 21	44	8.24	27
Right middle frontal gyrus	24, 42, 27	9	8.12	13
Right postcentral gyrus	54, -24, 45	2	7.98	99
Left lateral parietal sulcus	-27, -57, 66	7	7.98	17
Right fusiform gyrus	48, -57, 3	37	7.58	15
Right ventrolateral prefrontal cortex	42, 21, 15	44	7.54	22
Right cerebellum	9, -60, -27	-	7.07	22
Left cerebellum	-9, -69, -21	-	7.01	18

*p* < .005, 13-voxel extent. All coordinates MNI, organized by descending F-values.

We then conducted subsequent *t*-tests to identify the patterns of neural activity that were attributable to age (by identifying neural activity that was greater for YA than OA and also for OA than YA), and older adults’ global cognitive function (by identifying neural activity that was unique to OA with relatively preserved global cognitive function as compared to OA with relatively impaired global cognitive function, and vice versa). For the age comparison, we found that as compared to OA, YA had heightened activation in right cuneus (6, -90, 45) and thalamus (0, -6, 9). However, as compared to YA, OA had heightened activation in right middle frontal gryus (BA 9: 63, 15, 36), right ventrolateral prefrontal cortex (BA 45/46: 48, 27, 12), right superior frontal gyrus (BA 6: 6, 6, 60), and right parietal cortex (BA 7: 30, -60, 48). See Table 5 for complete list of activations.

**Table 5:** Statistically significant clusters from t-tests resulting from the main effect of group. —indicates a region that does not fall within a Brodmann area.

Contrast	Region	MNI Coordinates	Brodmann Area	T-value	Cluster size (mm^3^)
YA > OA	Cuneus	6, -90, 45	18	7.54	1466
	Left cerebellum	-30, -66, -45	-	3.97	45
	Thalamus	0, -6, 9	-	3.36	29
OA > YA	Right Superior Parietal Lobule	30, -60, 48	7	4.01	34
	Left Middle Temporal Gyrus	-54, -78, 21	19	3.9	86
	Right Ventrolateral prefrontal cortex	42, 6, 24	9	3.74	32
	Right Middle Frontal Gyrus	63, 15, 36	9	3.51	35
	Right Postcentral Gyrus	69, -18, 21	43	3.49	28
	Right Superior Frontal Gyrus	6, 6, 60	6	3.49	20
	Right Precentral Gyrus	69, -12, 39	6	3.41	40
	Left Postcentral Gyrus	-57, -24, 57	2	3.24	18
	Right Middle Occipital Gyrus	45, -81, 6	19	3.09	25
	Right Ventrolateral prefrontal cortex	48, 27, 12	45/46	2.86	19
OA with relatively preserved global cognitive function > OA with relatively impaired global cognitive function	Left fusiform gyrus	-30, -42, -12	37	4.75	34
	Left insula	-39, -3, 15	13	3.25	19
OA with relatively impaired global cognitive function > OA with relatively preserved global cognitive function	Left cerebellum	-21, -33, -42	-	4.72	385
	Right superior frontal gyrus	15, 0, 75	6	4.62	95
	Right cerebellum	15, -33, -39	-	4.20	98
	Right anterior cingulate gyrus	15, 24, 36	32	4.18	848
	Right ventrolateral prefrontal cortex	45, 30, -15	11/47	4.15	189
	Right dorsomedial prefrontal cortex	12, 39, 30	32	3.95	848
	Right inferior temporal gyrus	60, -39, -9	20	3.92	64
	Right cerebellum	45, -51, -21	-	3.73	77
	Right middle frontal gyrus	42, 48, -12	10/47	3.36	46
	Left ventrolateral prefrontal cortex	-24, 24, -18	11/47	3.34	39
	Right middle frontal gyrus	24, 45, 24	8/9	3.25	22

Next, we compared OA with relatively preserved to OA with relatively impaired global cognitive function. This analytical approach allowed us to identify patterns of neural activity that differed depending upon older adults’ global cognitive function. Here, we only found two peaks that were more active for OA with relatively preserved global cognitive function as compared to OA with relatively impaired global cognitive function: the left fusiform gyrus (BA 37: -30, -42, -12) and left insula (-39, -3, 15). See Table 5. OA with relatively impaired global cognitive function, however, had greater activation as compared to OA with relatively preserved global cognitive function in bilateral ventrolateral prefrontal cortex (right BA 11/47: 45, 30, -15; left BA 11/47: -24, 24, -18), right anterior cingulate gyrus (BA 32: 15, 24, 36), right dorsomedial prefrontal cortex (BA 32: 12, 39, 30), and right middle frontal gyrus (BA 8/9: 24, 45, 24). See Table 5.

Given our behavioral finding that the pity differential was associated with older adults’ pre-exiting attitudes toward homeless individuals and their global cognitive function (which was associated with their years of education), we also conducted a separate 2 X 3 whole-brain ANOVA with pre-existing attitudes and global cognitive function entered as covariates. The results were largely consistent between the two analyses. Importantly, all of the peaks of interest that were examined in the subsequent analyses (e.g., the left insula, anterior cingulate cortex, ventrolateral prefrontal cortex) were still significant at the corrected threshold in the contrast of interest (e.g., OA with relatively preserved cognitive function > OA with relatively impaired cognitive function).

#### Question 2: Parametric analysis: Are any of the regions from the main effect of group associated with pity?

The results from the whole brain ANOVA indicated that there were group differences in neural activity as a function of global cognitive function. However, they do not elucidate why those differences emerged. Specifically, it remains an open question to what extent the differences in activation were related to how much pity participants expressed to the not-responsible as compared to responsible homeless individuals, particularly since no main effect of stigma acquisition or interaction emerged in the whole-brain ANOVA.

In order to address this question, we conducted two separate analyses to better understand the differences that emerged in the whole-brain ANOVA: a whole-brain multiple regression (using group, the pity differential scores, and the interaction between the two) and three parametric modulations (one for each group) to identify the regions that were more active the more pity the individual participants indicated that they felt for each target. Each of these two approaches provided important insight into understanding why group differences emerged in neural activity in the whole-brain ANOVA. On the one hand, because the multiple regression identified neural regions that were more active as a function of participants’ pity differential, this analysis spoke to more general neural differences associated with attitude malleability. On the other hand, because the parametric analysis measures changes in brain activity based on participants’ trial-by-trial ratings of pity, it identified the neural regions that emerged from the group effect (see Table 5) that were associated with participants’ specific trial-by-trial decisions about pity.

For the regression, we identified neural regions that were more active in the group X pity differential interaction. The pity differential scores were centered around the mean. Here, we found heightened activation in the bilateral ACC and right vlPFC (see Table 6). Subsequent t-tests demonstrated that activity in these regions was higher for older adults with relatively impaired global cognitive function as compared to older adults with relatively preserved global cognitive function and young adults (who did not differ from each other). However, this effect only reached traditional levels of statistical significance in the left ACC (peak: -12, 45, 0).

**Table 6:** Statistically significant clusters resulting from the interaction between group and participants’ pity differential scores in a whole-brain multiple regression.

Region	MNI Coordinates	Brodmann area	T	cluster
Right Superior Temporal Gyrus	33, 18, -42	38	5.08	52
Right Precentral Gyrus	72, 0, 18	6	4.56	62
Left Anterior Cingulate	-12, 45, 0	32	4.43	97
Right Fusiform Gyrus	54, -6, -24	20	4.2	50
Right ACC/Medial Frontal Gyrus	12, 51, 6	10/24/32	4.36	77
Right Fusiform Gyrus	39, -33, -18	20	4.34	14
Right Uncus	21, -12, -30	28	4.15	25
Right Inferior Frontal Gyrus	15, 18, -21	47	4.1	57

For the parametric analysis, we created masks from the *t*-tests of the whole brain ANOVA (above) to isolate neural activation that was affected by age (young adults > OA with relatively preserved cognitive function) and global cognitive function (OA with relatively preserved global cognitive function > OA with relatively impaired global cognitive function, and OA with relatively impaired global cognitive function > OA with relatively preserved global cognitive function). We then used the inclusive mask created from these *t*-tests to identify overlapping regions that emerged from the parametric modulation analysis. (See Table 7 for individual group activations from the parametric modulation). This analysis allowed us to identify the regions from the main effect of group that were related to pity, but not other processes, such as differences in perceived valence and arousal ratings (because we controlled for individual differences in perceived valence and arousal in the parametric analysis; see fMRI data analysis section above).

**Table 7:** Statistically significant clusters resulting from the parametric modulation with pity ratings for all groups.

Group	Region	MNI Coordinates	Brodmann area	T	cluster
Young adults	Right cerebellum	9, -54, -3	-	3.90	15
OA with relatively preserved global cognitive function	Left superior temporal gyrus	-66, -27, 12	22	4.26	96
	Left fusiform gyrus	-48, -60, 6	37	4.51	96
	Left precentral gyrus	-39, -9, 33	6	4.18	46
	Left insula	-39, -6, 21	13	3.67	*
	Right posterior cingulate gyrus	18, -27, 36	31	4.07	17
	Left thalamus	-21, -21, 9	-	3.92	23
	Left cerebellum	-3, -57, -33	-	3.68	15
	Right orbitofrontal cortex	15, 18, -18	11/25	3.48	40
	Right superior temporal gyrus	57, -12, 9	22	3.44	17
	Right thalamus	6, -15, -6	.	3.41	17
	Right lateral parietal sulcus	18, -63, 51	7	3.40	63
OA with relatively impaired global cognitive function	Left superior temporal gyrus	-60, -27, 12	22	3.57	34
	Right middle frontal gyrus	21, 57, 9	10	3.41	15
	Right lingual gyrus	24, -87, 3	2	3.26	13

- Indicates a region that does not fall within a Brodmann area.

* Indicates a subthreshold cluster.

**Fig 1:**
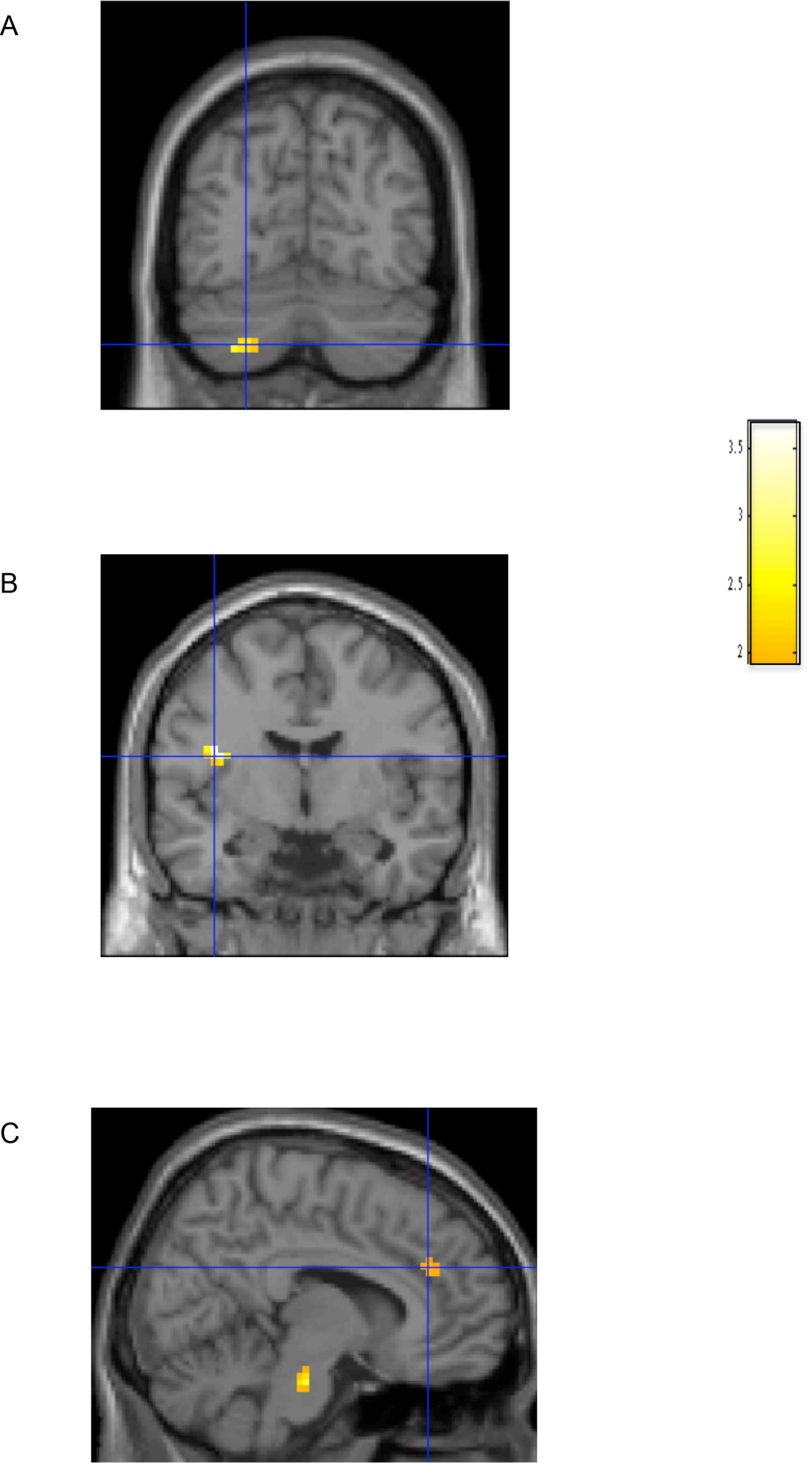
Results from the *t*-tests from the whole-brain ANOVA for YA > OA with relatively preserved cognitive function (A), OA with relatively preserved global cognitive function > OA with relatively impaired global cognitive function (B), and OA with relatively impaired global cognitive function > OA with relatively preserved global cognitive function (C) when masked with the unbiased parametric modulation for YA, OA with relatively preserved global cognitive function, and OA with relatively impaired global cognitive function, respectively. All data shown at *p* < .05 corrected on a T1 template brain from SPM8.

These analyses revealed that cerebellar activation tracked with pity ratings more strongly for YA than for OA with relatively preserved global cognitive function. Activation in the left insula (-39, -6, 21) tracked more strongly with pity ratings for OA with relatively preserved global cognitive function as compared to OA with relatively impaired global cognitive function. Activation in the right superior frontal gyrus (BA 8: 30, 45, 45), right ventrolateral prefrontal cortex (BA 11/47: 45, 30, -15), and right anterior cingulate cortex (BA 32: 12, 36, 27) tracked with pity ratings more strongly for OA with relatively impaired global cognitive function as compared to OA with relatively preserved global cognitive function. No other group differences in the parametric effect of pity were revealed. See Table 8, Fig 1.

**Table 8:** Statistically significant clusters resulting from the mask between the t-test and parametric results. —indicates a region that does not fall within a Brodmann area.

Group	Region	MNI Coordinates	Brodmann area	T	cluster
Young adults	Right cerebellum	33, -30, -48	-	3.68	31
	Right cerebellum	9, -54, -3	-	3.48	32
	Left cerebellum	-36, -39, -36	-	2.34	16
OA with relatively preserved global cognitive function	Left insula	-39, -6, 21	13	3.67	15
OA with relatively impaired global cognitive function	Right midbrain	12, -21, -27	-	2.87	29
	Right ventrolateral prefrontal cortex	45, 30, -15	11/47	2.82	26
	Right superior frontal gyrus	30, 45, 45	8	2.46	77
	Right anterior cingulate cortex	12, 36, 27	32	2.20	17

#### Question 3: Is the pity differential driven by empathy or cognitive control?

Our final goal was to determine whether reduced empathy (e.g., insula and anterior cingulate cortex) [17,18,19] or regulatory effort (e.g., right ventrolateral prefrontal cortex) [16, 17,19] was associated with the decreased pity differential we observed for older adults with relatively impaired global cognitive function. We took two separate approaches to provide converging evidence on this question. First, we entered OA group, stigma onset condition, and participants’ self-reported empathy scores (which were centered around the mean), into a whole-brain ANOVA. This allowed us to identify neural regions associated with self-reported empathy when controlling for group and stigma onset condition. Self-reported empathy was associated with increased activation in the left insula (BA 13: -33, -36, 18), whereas the interaction did not reveal heightened activation in the insula at our corrected threshold (see fMRI methods).

Our second approach involved creating independently defined ROIs by identifying peaks from the two regions commonly associated with empathy (e.g., left insula and left anterior cingulate cortex) [27] and the region identified in regulatory effort (right ventrolateral prefrontal cortex) using Neurosynth (http://neurosynth.org) [35]. We identified these peaks by using the search term “empathy” in the meta-analysis function on Neurosynth. We then identified the peaks from the reverse inference map with the highest z-scores in our areas of interest. This approach allowed us to identify the peaks in the insula and ACC (that had been identified in the studies on empathy) that were the most strongly associated with experiences of empathy, as opposed to peaks that are likely to be active in any cognitive task. See fMRI methods for more details. We identified the left insula peak (-48, 6, 4) and left anterior cingulate cortex peak (-4, 26, 26) for the ROI analyses by conducting a search on all studies associated with empathy (z-scores = 5.33 and 6.1, respectively). The right ventrolateral prefrontal cortex peak (50, 28, -8) had a z-score of 7.09.

We used these peaks to extract parameter estimates in the not-responsible and responsible conditions from the impression formation task. Of interest was whether the dissociation in neural activity in these regions predicted older adults’ pity differential score when, as with the behavioral analyses, we controlled for their pre-existing attitudes toward homeless individuals and participant years of education by conducting hierarchical linear regressions with attitudes and years of education entered into the first step (in that order), and neural activity entered into the second step. For the insula, results revealed that although the model was not significant at the first step: (*F*(2,29) = 2.78, *p* = .08), explicit attitudes did significantly predict pity differential (see Table 9). However, adding the insula activation in the second step significantly accounted for 31% of the variance in the pity differential score: (*F*(3,29) = 3.81, *p* = .02; Δ*F*(1,26) = 5.02, *p* = .03). A similar pattern emerged for the left anterior cingulate cortex. Here, adding the ACC activation in the second step accounted for 29% of the variance in the pity differential score: (*F*(3,29) = 3.46, *p* = .03; Δ*F*(1,26) = 4.17, *p* = .05). However rvlPFC activation did not predict the pity differential score when controlling for explicit attitudes and the pity differential score (*F*(3,29) = 2.40, *p* = .09; Δ*F*(1,26) = 1.51, *p* = .23). Regression statistics are reported in Table 9.

**Table 9:** Summary of Hierarchical Regression Analysis Predicting Older Adults’ Pity differential score from left insula/left anterior cingulate cortex (ACC).

Variable	ß	*t*	*R*	*R*^*2*^	Δ*R*^*2*^
Step 1			.41	.17	.
Explicit attitudes	-.41	-2.31*			
Years of education	.10	.59			
Step 2			.55/.53	.31/.29	.13/.12
Explicit attitudes	-.36/-.36	-2.16*/-2.15*			
Years of education	.05/.10	.30/.59			
L. Insula/ L. ACC	.37/.34	2.24*/2.04*			

* *p* ≤ .05.

## Discussion

There are two key findings from this study. First, although older adults’ attitudes toward stigmatized individuals are malleable, individual differences in older adults’ global cognitive function may reduce the extent to which they are malleable when controlling for individuals differences in explicit bias. Second, these differences were associated with heightened activation in the left insula and anterior cingulate cortex when older adults’ evaluated homeless individuals who were depicted as being not responsible as compared to responsible for the onset of their condition.

Our findings suggest that older adults with relatively impaired global cognitive function differ from older adults with relatively preserved global cognitive function in the extent to which they empathize with stigmatized individuals who are depicted as being not responsible for the onset of their condition. In the whole-brain ANOVA, we found group differences in activation in regions previously associated with empathy (e.g., the left insula and anterior cingulate cortex) [19] and regulatory effort (ventrolateral prefrontal cortex). Through two separate analyses, we found that the insula was associated with empathy. First, we found that older adults’ self-reported empathy scores uniquely predicted activity in the left insula. In a separate analysis, we independently defined peaks within these regions that were identified via meta-analyses as being involved in empathy. This analysis demonstrated that the ACC and insula predicted older adults’ pity differential, whereas regulatory effort did not. Finally, in the parametric analysis, we found heightened left insula activation for the older adults with relatively preserved global cognitive function, but heightened activity in the right anterior cingulate cortex for older adults with relatively impaired global cognitive function. Both were positively associated with the pity differential, suggesting that different processes underlie how the two groups of older adults evaluate outgroup members, which may inform their subsequent pity differential. In both cases, however, the greater extent to which they used regions associated with empathy, the more malleable their attitudes.

The results from the current study contribute to the growing body of work demonstrating that individual differences in cognitive ability are associated with older adults’ bias by demonstrating that these deficits also extend to attitude malleability. Importantly, reduced global cognitive function was associated with reduced attitude malleability (e.g., pity differential). However, these findings must be taken with caution. Although we did rule out some of the factors that could interact with global cognitive function to influence these results–notably years of education (which was strongly correlated with global cognitive function in our sample and has been linked to higher prejudice) [37]–others could underlie the effects observed here. For instance, socioeconomic status (which was not measured in our participant group), differences in strategies in performing the tasks, and race or ethnicity (which were not varied in the current task since participants were predominantly White), could exacerbate or underlie these results. Moreover, because we analyzed global cognitive function among older adults, and not younger cohorts, our results cannot determine whether the effects are specific to older adults or are more generally tied to differences in global cognitive function (i.e., among middle-aged or young adults). These results should therefore be interpreted with those caveats in mind.

That said, however, an important contribution of the current work is that the extent to which older adults’ attitudes are malleable is a distinct (albeit overlapping) construct from explicit bias. In our sample, attitude malleability was more strongly associated with empathy (as predicted by activation in the left insula and left anterior cingulate cortex) than it was with regulatory effort (as predicted by activation in the right vlPFC). Thus, it may be the case that the older adult participants with relatively impaired global cognitive function were less successful in using empathy to increase the malleability of their attitudes. Such an interpretation would be consistent with previous findings that cognitive aspects of empathy decline with age [24], but would add to these findings by suggesting that it may be reductions in global cognitive function, and not age per se, that is associated with these declines.

In the context of the current task, one possible explanation for the results we observed is that individual differences in global cognitive function may impact the manner in which older adults experience pity. Pity can stem from two disparate causes: feeling empathy–which involves the perceiver feeling the same emotions as the target (also known as perspective taking; [27])–or from feeling personal distress about the situation–which does not necessarily involve taking someone else’s feelings into account. The insula and the anterior cingulate cortex have both been implicated in empathic response to seeing other individuals experience pain [27,38]. Personal distress, however, appears to elicit a neural response in the ventrolateral prefrontal cortex and the anterior cingulate cortex [39,40]. It is important to note that this interpretation is speculative, and should be taken with caution. However, future research should examine whether available cognitive resources influence which route individuals may take to make similar subjective judgments of pity.

Another possibility is that the task required more cognitive effort for the OA with relatively impaired global cognitive function as compared to those with relatively preserved cognitive function. Prior research has demonstrated that some older adults have heightened activation in the prefrontal cortex when they perform cognitively demanding tasks to the same level as younger adults [41], and related research has suggested that older adults may recruit compensatory processes at lower levels of cognitive load than younger adults [42]. With respect to the current study, it is possible that OA with relatively impaired global cognitive function had higher levels of activity in the lateral prefrontal cortex during the task as compared to OA with relatively preserved global cognitive function because the task was more effortful for them to perform and required them to recruit compensatory processes. If this is the case, the task could have been more effortful for several different reasons (e.g., remembering the sentences that preceded each image required working memory, updating one’s impression of the target may have required inhibition of the original response). Future research should more closely examine these possibilities.

At first glance, it may seem surprising that older adults’ pity differential relied more on empathy than regulatory effort; one possibility is that this was due to the fact that we controlled for explicit bias in our analyses. Older adults’ bias has been widely attributed to declines in regulatory function [1,2,3,4,5], therefore the extent to which bias and attitude malleability are overlapping constructs may rely on regulatory effort, whereas empathy dissociates the latter from the former.

Although not the central focus of the present study, it is interesting to note that the insula was not more active for the young adults as compared to the older adults. Moreover, Krendl, Moran, and Ambady [19] found that the insula was one of the key regions that was more active for young adults when evaluating homeless individuals who were described as being not responsible for their condition as compared to those who were responsible for their condition. Young adults also reported feeling more pity for the former than they did for the latter. Together, this suggests that the two groups did not differ in their recruitment of empathy toward homeless individuals.

Unexpectedly, the results from the whole-brain ANOVA did not parallel the results from the parametric analysis. We had anticipated that the parametric analysis results would reveal similar regions to the whole-brain ANOVA, identifying activity that varied as an effect of stigma condition (responsible or not responsible). This assumption was based on the fact that, when younger adults were tested on a similar paradigm [19], the regions that showed greater activation for the not responsible as compared to the responsible condition were similar regions to those revealed in the parametric analysis in the current study. Specifically, insula activity was heightened in the prior study when perceivers evaluated a homeless individual who was described as not being responsible for becoming homeless, and insula activity tracked with pity in the present study. In the current study, we did find a main effect of stigma acquisition in the behavioral pity ratings, but not of group. However, in the fMRI results, we found an effect of group, but not stigma acquisition. Upon further examination of the data, it was apparent that this was because condition (responsible vs. not responsible) had different effects on neural activity in each of the three groups. It is worth noting that the results from the whole-brain ANOVA did parallel the results for the parametric analysis for both groups of older adults when examined at a slightly more liberal threshold. Those data were not reported in order to ensure we did not commit any Type I errors, yet they suggest that the patterns were somewhat consistent between the two analyses.

One of the limitations of the current study was that we did not include a baseline condition in the responsibility manipulation (e.g., a neutral responsibility condition). Although we did not include another condition due to concerns with participant fatigue and carryover effects from neighboring trials, such a condition could have provided important insight into the results observed in the current study, and should be considered in future research.

The findings of this study extend a small, but growing, body of literature that examines the malleability of neural networks in response to perceiving stigma [17,19,43]. Importantly, this is the first study to extend this line of research into the aging population. Together, our findings suggest that although the patterns of neural activity engaged by older adults toward outgroup members are malleable, this malleability does not necessarily improve the attitudes that older adults with relatively impaired global cognitive function express toward outgroup members.
